# Effects of hypoxia-inducible factor prolyl hydroxylase inhibitors on hemoglobin, B-type natriuretic peptide, and renal function in anemic heart failure patients: A systematic review and *meta*-analysis

**DOI:** 10.1016/j.ijcha.2025.101653

**Published:** 2025-03-22

**Authors:** Hidekatsu Fukuta, Toshihiko Goto, Takeshi Kamiya

**Affiliations:** a Core Laboratory, Nagoya City University Graduate School of Medical Sciences, Nagoya, Japan; b Department of Cardiology, Nagoya City University Graduate School of Medical Sciences, Nagoya, Japan; c Department of Medical Innovation, Nagoya City University Graduate School of Medical Sciences, Nagoya, Japan

**Keywords:** Anemia, Hypoxia-inducible factor prolyl hydroxylase inhibitors, Heart failure, Systematic review, Meta-analysis

## Abstract

**Background:**

Anemia is a common comorbidity in heart failure (HF) patients, often leading to worsened outcomes. Hypoxia-inducible factor prolyl hydroxylase (HIF-PH) inhibitors represent a novel approach for anemia management, yet their efficacy and safety in HF patients remain unclear. We aimed to conduct a systematic review and *meta*-analysis of studies on the effects of HIF-PH inhibitors on hemoglobin, N-terminal prohormone of brain natriuretic peptide (NT-proBNP), or estimated glomerular filtration rate (eGFR) in HF patients with chronic kidney disease (CKD).

**Methods and Results:**

The search of electronic databases identified four studies including 98 patients. Among the included studies, two were conducted prospectively, while two were retrospective in design. No studies were identified that compared HIF-PH inhibitors with erythropoiesis-stimulating agents or placebo. In cases of significant heterogeneity (I^2^ > 50 %), data were pooled using a random-effects model; otherwise, a fixed-effects model was used. In pooled analyses, hemoglobin levels increased at one (weighted mean difference [WMD]: 0.697 [0.473, 0.920] g/dL; P_fix_ < 0.001; I^2^ = 24 %) and three months (WMD: 0.760 [0.443, 1.076] g/dL; P_fix_ < 0.001; I^2^ = 31 %) after the use of HIF-PH inhibitors. NT-proBNP levels showed a modest decrease at one month but no significant changes at three months. eGFR remained unchanged, and no severe adverse events were reported.

**Conclusion:**

This *meta*-analysis suggests that HIF-PH inhibitors effectively improve anemia in HF patients with CKD without notable changes in renal or HF-related biomarkers. However, the small number of included studies and the absence of a comparator group underscore the need for cautious interpretation of the findings.

## 1 Introduction

Heart failure (HF) is a significant concern in both clinical practice and public health. Despite advancements in HF treatment, mortality rates remain high, with recent data indicating about 50 % mortality at 5 years [1]. Chronic kidney disease (CKD) commonly coexists with HF, and patients who have both conditions frequently also suffer from anemia [2]. In HF patients with anemia, limited oxygen delivery and impaired skeletal muscle oxygen utilization during physical activity lead to reduced functional capacity [3]. Numerous studies indicate that treating anemia with iron supplementation improves symptoms, functional capacity, and quality of life, and is associated with reduced HF-related hospitalizations in iron-deficient HF patients [[4], [5], [6], [7], [8]]. However, iron deficiency accounts for less than 30 % of anemia cases in HF patients, with most anemia stemming from other causes, such as reduced erythropoietin production due to kidney dysfunction and intrinsic bone marrow issues [3].

Erythropoiesis-stimulating agents (ESAs) have been evaluated for their effects in anemic HF patients with CKD across multiple randomized controlled trials (RCTs) [[9], [10], [11]]. Meta-analyses of these trials have shown that ESA treatment, compared to placebo, improves anemia and symptoms, but has a neutral effect on overall mortality and HF hospitalizations, while increasing the risk of thromboembolic events [12,13].

Hypoxia-inducible factor (HIF) prolyl hydroxylase (PH) inhibitors represent a new approach for anemia treatment [2,14]. These drugs function by stabilizing the HIF complex, thereby promoting natural erythropoietin production. HIF-PH inhibitors also support iron mobilization for bone marrow use. Compared to ESAs, HIF-PH inhibitors induce lower but steadier erythropoietin levels, which may lead to fewer cardiovascular side effects at similar hemoglobin levels.

Although several studies have examined the impact of HIF-PH inhibitors on anemia and HF severity in anemic patients with HF, findings have varied, likely due to limited sample sizes or differences in study design [[15], [16], [17], [18]]. Therefore, we conducted a systematic review and *meta*-analysis to assess the efficacy and safety of HIF-PH inhibitors in treating anemia among HF patients with CKD.

## 2 Methods

This systematic review and *meta*-analysis has been registered on the International Platform of Registered Systematic Review and Meta-analysis Protocols with registration number of INPLASY2024100062 (https://doi.org/10.37766/inplasy2024.10.0062). This study was performed according to the Preferred Reporting Items for Systematic Review and Meta-analysis (PRISMA) statement [19].

### 2.1 Search strategy

Studies examining the effects of HIF-PH inhibitors in HF patients published until October 31, 2024 were identified using PubMed, Web of Science, and Scopus. For search of the eligible studies, the following keywords and Medical Subject Heading were used: *hypoxia-inducible factor prolyl hydroxylase inhibitor(s),* r*oxadustat, daprodustat, vadadustat, molidustat, desidustat, enarodustat, and heart failure.*

Literature search was also conducted by manual screening of reference lists of relevant reviews and retrieved articles.

Two researchers (HF and TK) independently performed the literature search. We initially reviewed the titles and abstracts of each study, and if a study was considered relevant, we proceeded to read the full text. Disagreements were resolved by consensus.

### 2.2 Selection criteria

We screened articles based on the title and abstract using predefined inclusion and exclusion criteria.

Inclusion criteria:

1. Prospective and retrospective cohort studies or RCTs.
2. Studies specifically designed for HF patients with anemia and CKD.
3. Studies evaluating the administration of HIF-PH inhibitors.
4. Studies assessing at least one of the following outcomes: hemoglobin levels, N-terminal prohormone of brain natriuretic peptide (NT-proBNP) levels, estimated glomerular filtration rate (eGFR), cardiovascular death, all-cause death, or hospitalization for HF.

Exclusion criteria:

1. Case–control studies.
2. Studies that did not focus on HF patients.
3. Studies that included only CKD patients without HF.
4. Studies that included dialysis dependent CKD patients.
5. Studies that did not evaluate the effects of HIF-PH inhibitors.
6. Review articles, case reports, editorials, or conference abstracts.
7. Non-English articles.

### 2.3 Outcomes

The primary outcome of interest was the hemoglobin levels at one and three months after the use of HIF-PH inhibitors. The secondary outcomes of interest were NT-proBNP levels at one and three months and eGFR levels at three months after the use of HIF-PH inhibitors. Other outcomes of interest were: all-cause death, cardiovascular death, and thromboembolic events throughout the study duration; and drug discontinuation due to adverse events.

### 2.4 Data extraction

Two reviewers (HF and TK) independently extracted relevant data from retrieved studies, including author, study design, study time, number of participants, baseline characteristics, outcomes of interest, and information on the methodological quality.

### 2.5 Quality assessment

The quality of included studies was assessed using the revised and validated version of the Methodological Index for Non-Randomized Studies (MINORS) [20]. Two reviewers (HF and TK) independently assessed the quality of retrieved studies. Disagreements were resolved by consensus.

### 2.6 Statistical analysis

For single arm studies, pre- and post-intervention data was synthesized using the mean change from baseline, and the corresponding standard error was used to calculate pooled effect estimates. When the outcome was reported as the median (range and/or interquartile range), the mean and standard error were estimated as previously described [21]. If the outcome was measured on the same scale, the weighted mean difference (WMD) and 95 % confidence interval (CI) were calculated. Otherwise, the standardized mean difference and 95 % CI were calculated. For each outcome, heterogeneity was assessed using the I^2^ statistic; the I^2^ > 50 % was considered significant. When there was significant heterogeneity, the data was pooled using a random-effects model; otherwise, a fixed-effects model was used. NT-proBNP levels were natural log-transformed prior to analysis to normalize their distribution. The results are presented on the log-transformed scale. For primary outcome, a one-study-removed analysis was performed to assess the influence of any one particular study on the overall *meta*-analysis result. Additionally, publication bias was assessed graphically using a funnel plot and mathematically using Egger test. A sensitivity analysis was conducted to assess the robustness of the results for the changes in hemoglobin levels at three months. Subgroup analyses were performed separately for (1) studies limited to patients switched from ESAs and those including patients newly prescribed HIF-PH inhibitors (2) prospective and retrospective studies. Comprehensive Meta Analysis Software version 2 (Biostat, Englewood, NJ, USA) was used for analyses.

## 3 Results

### 3.1 Search results and characteristics

**Fig. 1:**
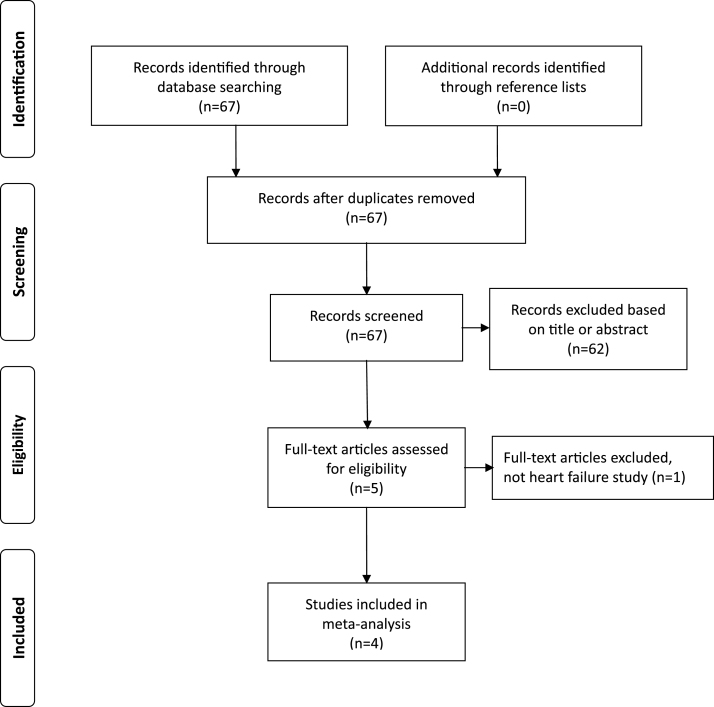
Selection process for studies included in *meta*-analysis.

The study identification and selection process is summarized in Fig. 1. We initially screened 67 studies through our systematic search strategy. Based on the title and abstract review, 62 studies were excluded for not meeting the selection criteria. Five studies were selected for full-text review, of which one was excluded because it focused only on patients with CKD [22]. The list of excluded study, along with the reasons for exclusion, is provided in Supplementary Table 1. Consequently, four studies including 98 patients were included. The study by Sezai et al. compared the effects of four different HIF-PH inhibitors. Therefore, the study was divided into four separate studies based on each HIF-PH inhibitor for this *meta*-analysis.

The characteristics of the included studies are presented in Table 1. Among the included studies, two were conducted prospectively, while two were retrospective in design. No studies were identified that compared HIF-PH inhibitors with ESAs or placebo. Regarding the patient populations, one study focused exclusively on patients switched from ESAs, two studies included both patients who switched from ESAs and those newly prescribed HIF-PH inhibitors, and one study examined only patients newly prescribed HIF-PH inhibitors. For the included studies, the MINORS scores ranged from 12 to 14, indicating a moderate risk of bias for non-comparative studies, where the maximum score is 16. Common issues identified were the absence of prospective calculation of the sample size and the retrospective nature of some studies. These biases influence the reliability and generalizability of the findings.

**Table 1:** Study characteristics.

Study	Design	Type of HIF-PH Inhibitors (initial dose)	Major inclusion criteria	No. of patients	Intervention period	Pre-ESA treatment	Primary and secondary outcomes (evaluation timing)	Quality
Nakamura 2023 [15]	Prospective, Single arm pre-post	Not available	Symptomatic HF and renal anemia	13	3 months	15 %	Hemoglobin (3 M), NT-proBNP (3 M), eGFR (3 M)	14
Yazaki 2024 [16]	Retrospective, Single arm pre-post	Vadadustat (300 mg/day)	Symptomatic HF and renal anemia	13	1 month	0 %	Hemoglobin (1 M), NT-proBNP (1 M)	12
Kambara 2024 [17]	Retrospective, Single arm pre-post	Roxadustat 59 %, Daprodustat 36 %, Vadadustat 5 %	Symptomatic HF and renal anemia	32	3 months	39 %	Hemoglobin (1 M, 3 M), NT-proBNP (1 M, 3 M)	12
Sezai 2024 [18]	Prospective, Single arm pre-post	Roxadustat (70 mg/day)	Symptomatic HF and renal anemia receiving CERA	10	6 months	100 %	Hemoglobin (1 M, 3 M), NT-proBNP (1 M, 3 M), eGFR (3 M)	14
Sezai 2024 [18]	Prospective, Single arm pre-post	Daprodustat (4 mg/day)	Symptomatic HF and renal anemia receiving CERA	10	6 months	100 %	Hemoglobin (1 M, 3 M), NT-proBNP (1 M, 3 M), eGFR (3 M)	14
Sezai 2024 [18]	Prospective, Single arm pre-post	Vadadustat (300 mg/day)	Symptomatic HF and renal anemia receiving CERA	10	6 months	100 %	Hemoglobin (1 M, 3 M), NT-proBNP (1 M, 3 M), eGFR (3 M)	14
Sezai 2024 [18]	Prospective, Single arm pre-post	Molidustat (25 mg/day)	Symptomatic HF and renal anemia receiving CERA	10	6 months	100 %	Hemoglobin (1 M, 3 M), NT-proBNP (1 M, 3 M), eGFR (3 M)	14

CERA indicates continuous erythropoietin receptor activator; eGFR, estimated glomerular filtration rate; ESA, erythropoiesis-stimulating agent; NT-proBNP, N-terminal prohormone of brain natriuretic peptide; HIF-PH, hypoxia-inducible factor prolyl hydroxylase; HF, heart failure.

1 M indicates one month after initiation of HIF-PH inhibitors and 3 M indicates three months after initiation of HIF-PH inhibitors.

The key characteristics of the included studies are summarized in Supplementary Tables 2 and 3, and the detailed descriptions of each study are provided below.

1 Nakamura et al. (2023) [15].

This prospective study included 13 patients with symptomatic HF and renal anemia (median age: 77 years, 54 % male). The median left ventricular ejection fraction (LVEF) was 48 %, with 31 % of patients having LVEF < 40 %. The most common etiology of HF was valvular heart disease (31 %), followed by dilated cardiomyopathy (23 %). The median hemoglobin level was 9.7 g/dL, NT-proBNP was 3082 pg/mL, and eGFR was 24.9 mL/min/1.73 m^2^. ESAs were switched to HIF-PH inhibitors in 15% of patients. After 3 months of treatment, hemoglobin significantly increased by 1.1 g/dL, NT-proBNP showed no significant change, and eGFR remained stable.

2 Yazaki et al. (2024) [16].

This retrospective study evaluated 13 patients with symptomatic HF and renal anemia (mean age: 75 years, 46 % male). The mean LVEF was 50 %, with 39 % of patients having valvular heart disease. The mean hemoglobin level was 9.7 g/dL, NT-proBNP was 4357 pg/mL, and eGFR was 29.4 mL/min/1.73 m^2^. All patients were newly initiated on vadadustat (starting dose: 300 mg/day) without prior ESA use. After 1 month of treatment, hemoglobin increased by 1.6 g/dL, and NT-proBNP decreased by 1990 pg/mL, both significantly, but eGFR remained unchanged.

3 Kambara et al. (2024)[17].

This retrospective study included 32 patients with HF and renal anemia (mean age: 82 years, 62 % male). The mean LVEF was not reported, but 16 % of patients had LVEF < 40 %. Ischemic heart disease was the most common etiology, followed by valvular heart disease (39 %). The mean hemoglobin level was 10.1 g/dL, NT-proBNP was 1177 pg/mL, and eGFR was 32.6 mL/min/1.73 m^2^. Patients were treated with roxadustat (59 %), daprodustat (36 %), or vadadustat (5 %). ESAs were switched to HIF-PH inhibitors in 38.5 % of patients. After 3 months, hemoglobin increased by 1.5 g/dL, and NT-proBNP remained unchanged.

4 Sezai et al. (2024) [18].

This prospective study analyzed 40 patients, all of whom were switched from continuous erythropoietin receptor activator to HIF-PH inhibitors. The study included four subgroups receiving roxadustat (70 mg/day), daprodustat (4 mg /day), vadadustat (300 mg/day), or molidustat (25 mg/day). The mean age ranged from 80 to 84 years, and 70 % were male across all groups. The mean LVEF was not available, but 10–30 % of patients had LVEF < 40 %. The most common etiology of HF was ischemic heart disease (30–50 % depending on subgroup). The median hemoglobin levels ranged from 11.9 to 12.7 g/dL, NT-proBNP levels ranged from 886 to 1292 pg/mL, and eGFR ranged from 27.2 to 33.0 mL/min/1.73 m^2^ depending on the treatment group. After 3 months, hemoglobin increased by 0.6–1.2 g/dL, with no significant change in NT-proBNP or eGFR.

Regarding individual study limitations, all included studies did not compare outcomes with ESAs or placebo. In particular, all patients in the study by Sezai et al. were switched from continuous erythropoietin receptor activator to HIF-PH inhibitors, which may influence the interpretation of treatment effects. Individual study limitations include small sample sizes (Nakamura et al. and Yazaki et al. had only 13 patients), short follow-up duration (Yazaki et al. only 1 month), and potential heterogeneity due to the inclusion of multiple HIF-PH inhibitors (Kambara et al.).

### 3.2 Primary outcome

**Fig. 2:**
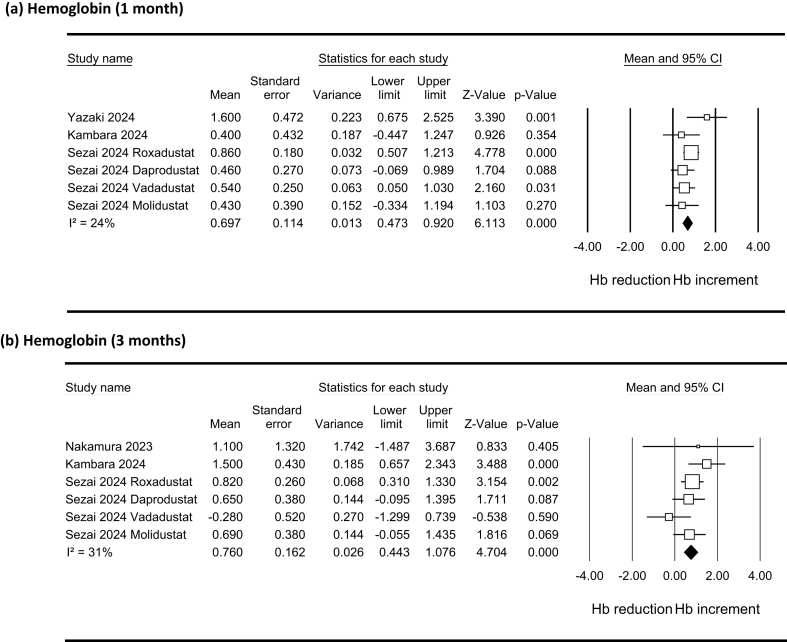
Forest plots showing the effects of hypoxia-inducible factor prolyl hydroxylase inhibitors on hemoglobin (Hb) levels (g/dL) at one (a) and three (b) months.

The effects of HIF-PH inhibitors on hemoglobin levels are shown in Fig. 2. Hemoglobin levels increased at one (WMD [95 % CI]: 0.697 [0.473, 0.920] g/dL; P_fix_ < 0.001; I^2^ = 24 %) and three months (0.760 [0.443, 1.076] g/dL; P_fix_ < 0.001; I^2^ = 31 %) after the use of HIF-PH inhibitors. No evidence of publication bias was found either in visual inspection of funnel plots or using the Egger test (P > 0.8; Supplemental Fig. 1). A one-study-removed analysis showed that none of the individual study significantly influenced the pooled estimate for the effect of HIF-PH inhibitors on hemoglobin levels (Supplemental Fig. 2).

### 3.3 Secondary and other outcomes

**Fig. 3:**
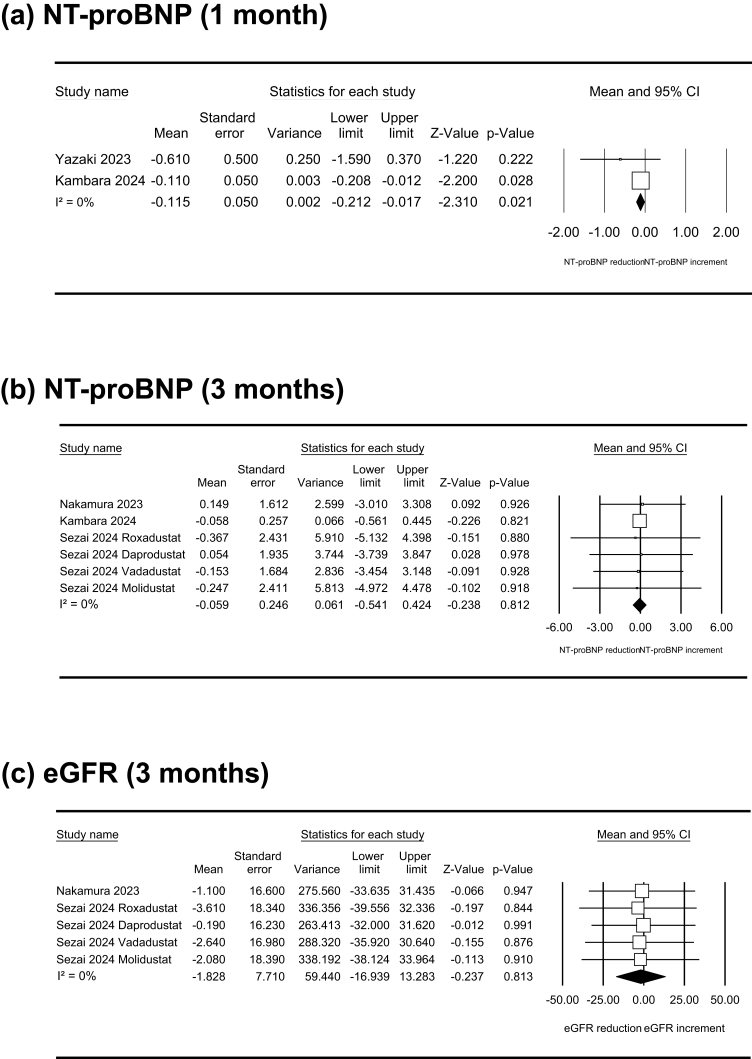
Forest plots showing the effects of hypoxia-inducible factor prolyl hydroxylase inhibitors on N-terminal prohormone of brain natriuretic peptide (NT-proBNP) levels ln(pg/ml) at one (a) and three (b) months and on eGFR levels (mL/min/1.73 m^2^) at three months (c).

The effects of HIF-PH inhibitors on NT-proBNP and eGFR levels are shown in Fig. 3. NT-proBNP levels showed a modest decrease at one month (WMD [95 % CI]: −0.115 [-0.212, −0.017] ln[pg/ml]; P_fix_ < 0.05; I^2^ = 0 %), which corresponds to an approximate 10.9 % reduction in the original scale. However, no significant changes were observed at three months (−0.059 [-0.541, 0.424] ln[pg/ml]; P_fix_ = 0.81; I^2^ = 0 %). eGFR levels did not change at three months (WMD [95 % CI]: −1.828 [-16.939, 13.283] mL/min/1.73 m^2^; P_fix_ = 0.81; I^2^ = 0 %).

No major events, such as all-cause death, cardiovascular death, or thromboembolic events, were reported across the included studies. However, drug discontinuation was reported in one patient due to headache and hot flushes, and in another patient due to hospitalization for worsening HF.

### 3.4 Sensitivity analysis

When the analysis was performed separately for studies limited to patients switched from ESAs and those including patients newly prescribed HIF-PH inhibitors, hemoglobin levels significantly increased at three months after the use of HIF-PH inhibitors (WMD [95 % CI]: 0.630 [0.285, 0.975] g/dL; P_fix_ < 0.001; I^2^ = 17 %, and 1.462 [0.660, 2.263] g/dL; P_fix_ < 0.001; I^2^ = 0 %, respectively). When the analysis was performed separately for prospective and retrospective studies, hemoglobin levels significantly increased at three months after the use of HIF-PH inhibitors (WMD [95 % CI]: 0.638 [0.297, 0.980] g/dL; P_fix_ < 0.001; I^2^ = 0 %, and 1.500 [0.657, 2.343] g/dL; P_fix_ < 0.001; I^2^ = 0 %, respectively).

## 4 Discussion

The present *meta*-analysis showed that HIF-PH inhibitors significantly improved hemoglobin levels in HF patients with renal anemia. The robustness of the findings on hemoglobin levels was confirmed through sensitivity analyses. The one-study-removed analysis revealed that no single study significantly influenced the pooled estimates of hemoglobin changes at both one and three months. It should be noted that the study by Sezai et al. [18] was a RCT that evaluated four different HIF-PH inhibitors in separate patient groups. Each treatment group was independently randomized and analyzed, and thus treated as separate datasets in our *meta*-analysis. This methodological approach follows established methods for incorporating multi-arm RCTs into *meta*-analyses and allows for the evaluation of each treatment while minimizing potential bias [23]. However, no notable changes were observed in NT-proBNP or eGFR, key indicators related to HF severity and renal function, respectively. This suggests that while HIF-PH inhibitors are effective for managing anemia, their benefits may be limited in terms of directly improving HF severity or kidney function at least short-term follow-up.

This *meta*-analysis aligns with prior research on HIF-PH inhibitors. The PRO_2_TECT trial showed that vadadustat was effective in treating anemia in patients with non-dialysis-dependent CKD [24]. However, it failed to demonstrate noninferiority in cardiovascular safety compared to darbepoetin alfa, as the risk of major adverse cardiovascular events was higher than the predefined acceptable margin. Notably, these results remained consistent even in the subgroup analysis of symptomatic HF patients. Furthermore, vadadustat did not show significant benefits in renal outcomes compared to darbepoetin alfa, with similar rates of CKD progression observed in both treatment groups. These findings reinforce that while HIF-PH inhibitors improve anemia, they may not provide broader benefits for renal or cardiovascular outcomes.

HIF-PH inhibitors increase hemoglobin by stabilizing hypoxia-inducible factors, which in turn stimulate erythropoietin production [2,14]. This mechanism mimics the body’s natural response to low oxygen levels and may also trigger pathways related to iron regulation and angiogenesis. However, while HIF-PH inhibitors are effective in improving hemoglobin levels, their impact on renal function and cardiovascular outcomes is less clear. CKD progression and HF involve complex processes such as chronic inflammation, fibrosis, and vascular remodeling that are not directly addressed by erythropoietin production alone [25]. Furthermore, although HIF-PH inhibitors may increase vascular endothelial growth factor (VEGF), which could theoretically benefit hypoxic tissues [14], excessive VEGF activation may also lead to increased vascular permeability and inflammation, which could offset potential cardiovascular benefits.

The results of this *meta*-analysis indicate that while HIF-PH inhibitors are effective for managing anemia in patients with HF and CKD, they could be considered as an adjunct to established therapies to optimize patient outcomes. Clinicians might explore incorporating HIF-PH inhibitors into comprehensive treatment plans that include agents like sodium-glucose cotransporter 2 (SGLT2) inhibitors [[26], [27], [28]] and renin-angiotensin system (RAS) blockers [[29], [30], [31]], which address the multifaceted pathophysiology of CKD and HF by reducing intraglomerular pressure and cardiac workload. While anemia is a known contributor to HF symptom burden and may be effectively managed with HIF-PH inhibitors, these agents cannot be expected to fully replace the central role of established therapies like SGLT2 inhibitors and RAS blockade, which remain the cornerstone of effective HF and CKD management.

HIF-PH inhibitors offer a novel mechanism of anemia management by stimulating endogenous erythropoietin production without requiring exogenous ESAs[2,14]. This reduces the risk of excessive erythropoiesis and associated complications, such as hypertension and thromboembolic events, which have been concerns with traditional ESAs [12,13]. One potential advantage of HIF-PH inhibitors is their ability to enhance iron metabolism and reduce hepcidin levels, thereby improving iron availability for erythropoiesis. Additionally, these agents have been hypothesized to exert cardioprotective effects by inducing adaptive metabolic changes and reducing oxidative stress under hypoxic conditions. However, clinical data on these potential benefits remain limited. The expectation of fewer cardiovascular side effects is based on the observation that HIF-PH inhibitors lead to a more physiological increase in erythropoietin levels, unlike ESAs, which cause supraphysiological spikes that may contribute to cardiovascular events.

Despite these advantages, concerns remain regarding potential off-target effects, including alterations in angiogenesis and vascular homeostasis [14]. Long-term cardiovascular safety of HIF-PH inhibitors remains uncertain, and further trials, such as the ongoing study [32], will be critical in assessing their full risk–benefit profile.

This *meta*-analysis has several limitations that must be considered. First, the number of included studies was limited, and the sample sizes were small. Furthermore,　all included studies were single arm and did not compare to ESAs or placebo. Additionally, the lack of long-term cardiovascular and renal outcome data limits our understanding of the full risk–benefit profile of HIF-PH inhibitors. Second, all included studies were conducted in Japan, which may limit the generalizability of our findings to other populations. Currently, there are no published studies from other countries that meet our inclusion criteria. Given potential differences in patient demographics, medical practices, and genetic backgrounds, further research from diverse geographic regions is needed to validate our findings. Third, the moderate MINORS scores (12–14 out of 18) for the non-comparative cohort studies highlight several methodological limitations, including the absence of prospective calculation of the sample size and the retrospective nature of some studies. These biases underscore the need for cautious interpretation of the findings. Fourth, heterogeneity in patient populations across included studies could affect the generalizability of our findings. Finally, without direct comparisons among different HIF-PH inhibitors, it remains unclear whether certain agents may have distinct advantages. These limitations highlight the need for further research to clarify the role of HIF-PH inhibitors in comprehensive HF and CKD management.

## 5 Conclusion

This *meta*-analysis suggests that HIF-PH inhibitors improve anemia in HF and CKD patients but do not have a significant impact on NT-proBNP levels, a biomarker of HF severity, or renal function at least in short-term follow-up. While HIF-PH inhibitors may be a valuable addition to anemia management strategies, their application should be carefully considered alongside standard therapies for HF and CKD. Further research is needed to better understand the long-term effects and optimal use of HIF-PH inhibitors in more diverse HF patient populations.

**Grant supporting this paper.**

This paper is not funded by any external source.

**Systematic review registration****:**INPLASY2024100062.

## CRediT authorship contribution statement

**Hidekatsu Fukuta:** Writing – review & editing, Writing – original draft, Methodology, Data curation, Conceptualization. **Toshihiko Goto:** Writing – review & editing, Conceptualization. **Takeshi Kamiya:** Writing – review & editing, Data curation.

## Declaration of competing interest

The authors declare that they have no known competing financial interests or personal relationships that could have appeared to influence the work reported in this paper.

## References

[1] Roger V.L. (2021). Epidemiology of heart failure: a contemporary perspective. Circ. Res..

[2] McCullough P.A. (2011). Anemia of cardiorenal syndrome. Kidney Int. Suppl..

[3] Tang Y.D., Katz S.D. (2006). Anemia in chronic heart failure: prevalence, etiology, clinical correlates, and treatment options. Circulation.

[4] Okonko D.O., Grzeslo A., Witkowski T. (2008). Effect of intravenous iron sucrose on exercise tolerance in anemic and nonanemic patients with symptomatic chronic heart failure and iron deficiency FERRIC-HF: a randomized, controlled, observer-blinded trial. J. Am. Coll. Cardiol..

[5] Anker S.D., Comin Colet J., Filippatos G. (2009). Ferric carboxymaltose in patients with heart failure and iron deficiency. N. Engl. J. Med..

[6] Beck-da-Silva L., Piardi D., Soder S. (2013). IRON-HF study: a randomized trial to assess the effects of iron in heart failure patients with anemia. Int. J. Cardiol..

[7] Ponikowski P., van Veldhuisen D.J., Comin-Colet J. (2015). Beneficial effects of long-term intravenous iron therapy with ferric carboxymaltose in patients with symptomatic heart failure and iron deficiencydagger. Eur. Heart J..

[8] Jankowska E.A., Tkaczyszyn M., Suchocki T. (2016). Effects of intravenous iron therapy in iron-deficient patients with systolic heart failure: a meta-analysis of randomized controlled trials. Eur. J. Heart Fail..

[9] Swedberg K., Young J.B., Anand I.S. (2013). Treatment of anemia with darbepoetin alfa in systolic heart failure. N. Engl. J. Med..

[10] van Veldhuisen D.J., Dickstein K., Cohen-Solal A. (2007). Randomized, double-blind, placebo-controlled study to evaluate the effect of two dosing regimens of darbepoetin alfa in patients with heart failure and anaemia. Eur. Heart J..

[11] Ghali J.K., Anand I.S., Abraham W.T. (2008). Randomized double-blind trial of darbepoetin alfa in patients with symptomatic heart failure and anemia. Circulation.

[12] Kang J., Park J., Lee J.M. (2016). The effects of erythropoiesis stimulating therapy for anemia in chronic heart failure: a meta-analysis of randomized clinical trials. Int. J. Cardiol..

[13] Zhang H., Zhang P., Zhang Y. (2016). Effects of erythropoiesis-stimulating agents on heart failure patients with anemia: a meta-analysis. Postepy Kardiol Interwencyjnej.

[14] Gupta N., Wish J.B. (2017). Hypoxia-inducible factor prolyl hydroxylase inhibitors: a potential new treatment for anemia in patients with CKD. Am. J. Kidney Dis..

[15] Nakamura M., Imamura T., Sobajima M., Kinugawa K. (2023). Initial experience of hypoxia-inducible factor prolyl hydroxylase inhibitors in patients with heart failure and renal anemia. Heart Vessels.

[16] Yazaki M., Nabeta T., Takigami Y. (2024). Efficacy of hypoxia-inducible factor prolyl hydroxylase inhibitor on clinical parameters in patients with heart failure. Medicina (Kaunas).

[17] Kambara T., Shibata R., Sakamoto Y. (2024). Impact of HIF prolyl hydroxylase inhibitors in heart failure patients with renal anemia. BMC. Res. Notes.

[18] Sezai A., Abe M., Maruyama T. (2024). A prospective randomized controlled clinical study to investigate the efficacy and safety of hypoxia-inducible factor-prolyl hydroxylase inhibitors in non-dialysis patients with chronic heart failure and renal anemia switched from continuous erythropoietin receptor activator treatment. J. Clin. Med..

[19] Moher D., Liberati A., Tetzlaff J. (2009). Preferred reporting items for systematic reviews and meta-analyses: the PRISMA statement. Ann. Intern. Med..

[20] Slim K., Nini E., Forestier D. (2003). Methodological index for non-randomized studies (minors): development and validation of a new instrument. ANZ J. Surg..

[21] Hozo S.P., Djulbegovic B., Hozo I. (2005). Estimating the mean and variance from the median, range, and the size of a sample. BMC Med. Res. Method..

[22] Barratt J., Dellanna F., Portoles J. (2023). Safety of roxadustat versus erythropoiesis-stimulating agents in patients with anemia of non-dialysis-dependent or incident-to-dialysis chronic kidney disease: pooled analysis of four phase 3 studies. Adv. Ther..

[23] JPT. Higgins, J. Thomas, J. Chandler *et al.* Cochrane Handbook for Systematic Reviews of Interventions. 2019.

[24] Chertow G.M., Pergola P.E., Farag Y.M.K. (2021). Vadadustat in patients with anemia and non-dialysis-dependent CKD. N. Engl. J. Med..

[25] Brown N.J. (2013). Contribution of aldosterone to cardiovascular and renal inflammation and fibrosis. Nat. Rev. Nephrol..

[26] McMurray J.J.V., Solomon S.D., Inzucchi S.E. (2019). Dapagliflozin in patients with heart failure and reduced ejection fraction. N. Engl. J. Med..

[27] Anker S.D., Butler J., Filippatos G. (2021). Empagliflozin in heart failure with a preserved ejection fraction. N. Engl. J. Med..

[28] Heerspink H.J.L., Stefansson B.V., Correa-Rotter R. (2020). Dapagliflozin in patients with chronic kidney disease. N. Engl. J. Med..

[29] Brenner B.M., Cooper M.E., de Zeeuw D. (2001). Effects of losartan on renal and cardiovascular outcomes in patients with type 2 diabetes and nephropathy. N. Engl. J. Med..

[30] Lewis E.J., Hunsicker L.G., Clarke W.R. (2001). Renoprotective effect of the angiotensin-receptor antagonist irbesartan in patients with nephropathy due to type 2 diabetes. N. Engl. J. Med..

[31] McMurray J.J., Packer M., Desai A.S. (2014). Angiotensin-neprilysin inhibition versus enalapril in heart failure. N. Engl. J. Med..

[32] Iso T., Matsue Y., Mizukami A. (2022). Daprodustat for anaemia in patients with heart failure and chronic kidney disease: a randomized controlled study. ESC Heart Fail.

