# Supplementary figures and images for: Effects of hypoxia-inducible factor prolyl hydroxylase inhibitors on hemoglobin, B-type natriuretic peptide, and renal function in anemic heart failure patients: A systematic review and meta-analysis
Source: Int J Cardiol Heart Vasc. 2025 Mar 22;58:101653. doi: 10.1016/j.ijcha.2025.101653 (PMC11979936; doi:10.1016/j.ijcha.2025.101653)

## Slide 1
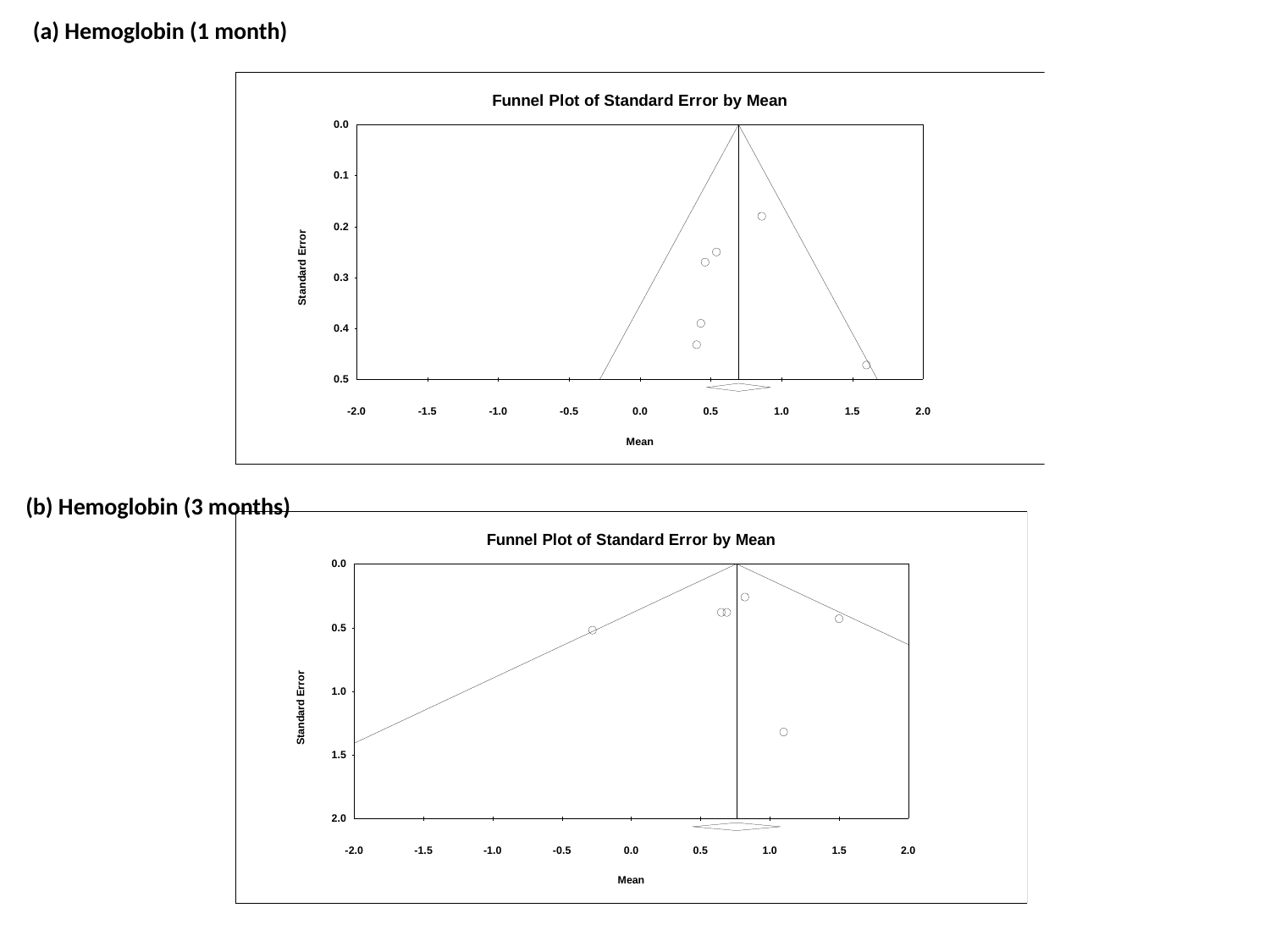

(a) Hemoglobin (1 month)
(b) Hemoglobin (3 months)

Supplement: Supplementary Data 2 [file mmc2.pptx]

## Slide 1
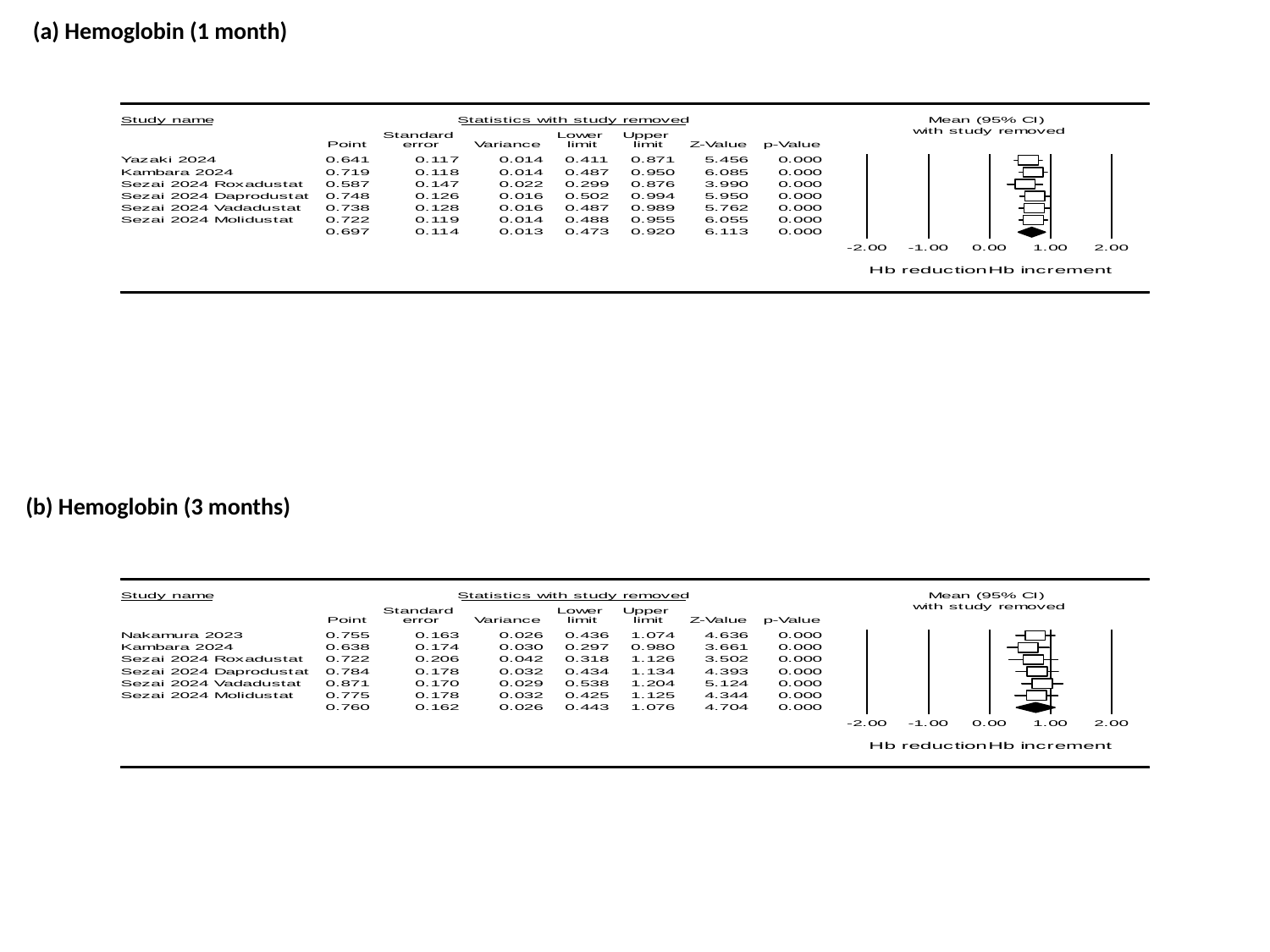

(a) Hemoglobin (1 month)
(b) Hemoglobin (3 months)

Supplement: Supplementary Data 3 [file mmc3.pptx]
