# Supplementary material for: Effects of hypoxia-inducible factor prolyl hydroxylase inhibitors on hemoglobin, B-type natriuretic peptide, and renal function in anemic heart failure patients: A systematic review and meta-analysis
Source: Int J Cardiol Heart Vasc. 2025 Mar 22;58:101653. doi: 10.1016/j.ijcha.2025.101653 (PMC11979936; doi:10.1016/j.ijcha.2025.101653)
Supplement: Supplementary Data 4 [file mmc4.docx]

**Supplementary Table 1.** List of excluded study and reasons for exclusion

| Study | Reasons |
| --- | --- |
| Barratt et al. (2023) Safety of Roxadustat Versus Erythropoiesis-Stimulating Agents in Patients with Anemia of Non-dialysis-Dependent or Incident-to-Dialysis Chronic Kidney Disease: Pooled Analysis of Four Phase 3 Studies. Advances in Therapy 2023; 40:1546-1559. | The study populations targeted CKD patients rather than heart failure patients. |
