# Supplementary material for: Effects of hypoxia-inducible factor prolyl hydroxylase inhibitors on hemoglobin, B-type natriuretic peptide, and renal function in anemic heart failure patients: A systematic review and meta-analysis
Source: Int J Cardiol Heart Vasc. 2025 Mar 22;58:101653. doi: 10.1016/j.ijcha.2025.101653 (PMC11979936; doi:10.1016/j.ijcha.2025.101653)
Supplement: Supplementary Data 5 [file mmc5.docx]

**Supplementary Table 2.** Patient characteristics

| Study | Age, years | NYHA  class≥Ⅲ | Men | EF, % | EF<40% | Valvular  heart  disease | Dilated  cardiomyopathy | Ischemic  heart  disease | AF | ACE-I,  ARB,  or ARNI | Beta-  blockers | Loop  diuretics |
| --- | --- | --- | --- | --- | --- | --- | --- | --- | --- | --- | --- | --- |
| Nakamura  2023 | 77 (74, 87) | 62% | 54% | 48 (34, 63) | NA | 31% | 23% | 15% | 23% | 100% | 85% | 69% |
| Yazaki  2024 | 75 ± 5 | 62% | 46% | 50 ± 14 | NA | 39% | 8% | 46% | 15% | 77% | 77% | 62% |
| Kambara  2024 | 82 ± 6 | NA | 62% | NA | 16% | NA | NA | 44% | 39% | 72% | 39% | 75% |
| Sezai  2024  Roxadustat | 81 (79–88) | NA | 70% | NA | 30% | 40% | 0% | 40% | NA | 75% | 75% | 90% |
| Sezai  2024  Daprodustat | 83 (77–88) | NA | 60% | NA | 20% | 30% | 20% | 30% | NA | 70% | 70% | 80% |
| Sezai  2024  Vadadustat | 80 (74–85) | NA | 70% | NA | 30% | 30% | 0% | 50% | NA | 80% | 70% | 80% |
| Sezai  2024  Molidustat | 84 (78–88) | NA | 70% | NA | 10% | 30% | 20% | 30% | NA | 75% | 75% | 85% |

Values are expressed as mean ± SD, median (interquartile range), or percentage of the group.

ACE-I indicates angiotensin converting enzyme inhibitor; AF, atrial fibrillation; ARB, angiotensin receptor blocker; ARNI, angiotensin receptor-neprilysin inhibitor; EF, ejection fraction; NA, not available; NYHA, New York Heart Association.

.
