# Supplementary material for: Effects of hypoxia-inducible factor prolyl hydroxylase inhibitors on hemoglobin, B-type natriuretic peptide, and renal function in anemic heart failure patients: A systematic review and meta-analysis
Source: Int J Cardiol Heart Vasc. 2025 Mar 22;58:101653. doi: 10.1016/j.ijcha.2025.101653 (PMC11979936; doi:10.1016/j.ijcha.2025.101653)
Supplement: Supplementary Data 6 [file mmc6.docx]

**Supplementary Table 3.** Patient characteristics

| Study | Hemoglobin, g/dL | NT-proBNP, pg/ml | eGFR, mL/min/1.73m^2^ |
| --- | --- | --- | --- |
| Nakamura 2023 | 9.7 (9.1–10.7) | 3082 (1108–5353) | 24.9 (20.1–35.9) |
| Yazaki 2024 | 9.7 ± 1.1 | 4357 (2651–15182) | 29.4 ± 10.6 |
| Kambara 2024 | 10.1 ± 1.4 | 1177 (642–1840) | 32.6 ± 11.6 |
| Sezai Roxadustat 2024 | 12.7 (11.4–13.0) | 1292.3 (461.4–3166.1) | 32.8 (22.1–36.6) |
| Sezai Daprodusta 2024 | 12.3 (11.9–13.1) | 1252.4 (430.9–1897.7) | 32.3 (21.9–34.3) |
| Sezai Vadadustat 2024 | 11.9 (11.4–12.4) | 1206.1 (866.6–3216.9) | 27.2 (20.4–34.8) |
| Sezai Molidustat 2024 | 12.3 (11.6–13.2) | 886.3 (388.1–2738.1) | 33.0 (26.2–40.9) |

Values are expressed as mean ± SD or median (interquartile range).

eGFR indicates estimated glomerular filtration rate; NT-proBNP, N-terminal prohormone of brain natriuretic peptide.
